# Rehabilitation interventions for persons with hip fracture and cognitive impairment: A scoping review

**DOI:** 10.1371/journal.pone.0273038

**Published:** 2022-08-15

**Authors:** Lauren Cadel, Kerry Kuluski, Walter P. Wodchis, Kednapa Thavorn, Sara J. T. Guilcher

**Affiliations:** 1 Leslie Dan Faculty of Pharmacy, University of Toronto, Toronto, Ontario, Canada; 2 Institute for Better Health, Trillium Health Partners, Mississauga, Ontario, Canada; 3 Institute of Health Policy, Management and Evaluation, University of Toronto, Toronto, Ontario, Canada; 4 Ottawa Hospital Research Institute, Ottawa, Ontario, Canada; 5 School of Epidemiology and Public Health, Ottawa, Ontario, Canada; 6 Rehabilitation Sciences Institute, University of Toronto, Toronto, Ontario, Canada; University Hospital Zurich, SWITZERLAND

## Abstract

**Background:**

Hip fractures are common fall-related injuries, with rehabilitation and recovery often complicated by cognitive impairment. Understanding what interventions exist, and in what settings, for people with hip fracture and co-occurring cognitive impairment is important in order to provide more evidence on rehabilitation and related outcomes for this population.

**Objective:**

To examine the extent, nature, and range of literature on rehabilitation interventions for adults with hip fracture and cognitive impairment.

**Methods:**

Articles were required to: include an adult population with hip fracture and cognitive impairment, include a rehabilitation intervention, and be published between January 1, 2000 and November 19, 2021. Articles were excluded if they were opinion pieces, study protocols, conference abstracts, or if they did not describe the rehabilitation intervention. Relevant articles were searched on the following electronic databases: MEDLINE, EMBASE, CINAHL Plus, APA PsycINFO, Cochrane Library, Web of Science, and the Physiotherapy Evidence Database. All articles were double-screened by two reviewers and disagreements were resolved through consensus. Data were extracted and synthesized using descriptive approaches.

**Results:**

Seventeen articles were included in this scoping review. We identified a variety of interventions targeting this population; about half were specific to physical rehabilitation, with the other half incorporating components that addressed multiple aspects of the care journey. Interventions had varying outcomes and no studies qualitatively explored patient or family experiences. All intervations were initiated in hospital, with less than half including cross-sectoral components. About half of the articles described modifying or tailoring the intervention to the participants’ needs, but there was limited information on how to adapt rehabilitation interventions for individuals with cognitive impairment.

**Conclusions:**

More work is need to better understand patient, family, and provider experiences with rehabilitation interventions, how to tailor interventions for those with cognitive impairment, and how to successfully implement sustainable interventions across sectors.

## Introduction

Globally, the proportion of the population that are older continues to rise, which presents a number of challenges for healthcare systems due to the increase in individuals with chronic conditions, disability, and injury [1]. Hip fractures are one of the most common fall-related injuries among older adults that result in hospitalization [2, 3].

Following a hip fracture, individuals frequently experience functional decline, morbidity, and institutionalization [4–7]. Hip fractures are also associated with high rates of acute readmissions, longer inpatient length of stays, and multimorbidity [8]. Hip fracture treatment and recovery can be further complicated by cognitive conditions, including delirium and/or dementia. Impaired cognition has been connected to an increased risk of falls and fall-related fractures (i.e., hip fractures) [9] due to an altered gait [10]. The proportion of patients with hip fracture who have dementia is nearly 20% in Canada [11], 13% in the United States [12, 13], and approximately 25% in the United Kingdom [14]. A systematic review including international literature reported that 24% of older patients with hip fracture experience delirium [15]. Importantly, persons with dementia and hip fracture often have worse short and long-term outcomes, including postoperative mortality at 1 month, 6 months, 12 months, and more than 12 months [16]. These differences in outcomes, are in part, due to differential pathways of care such that persons with dementia and hip fracture may not receive adequate rehabilitation for their care needs [17–19].

According to quality standards for hip fracture care in Canada, the United States, and the United Kingdom, all individuals who experience a hip fracture should receive a baseline cognitive assessment at admission and rehabilitation delivered by an interdisciplinary team (including those with cognitive impairment) [20–22]. Rehabilitation has demonstrated positive outcomes post-hip fracture, with improvements in functional status, mobility, balance, leg strength, health status, and social functioning [21, 22]. Additionally, rehabilitation interventions have shown promising outcomes for individuals with hip fracture and cognitive impairment [23]. For example, a systematic review of rehabilitation interventions for older adults with dementia and hip fracture identified that those with mild or moderate dementia showed similar improvements to those without dementia with respect to fall risk, ambulation, and function [23].

Despite these positive outcomes, current rehabilitation programs often exclude persons with hip fracture and co-occurring cognitive impairment [17–19]. As evidenced by systematic reviews conducted by Hebert-Davies et al. and Mundi et al., individuals with cognitive impairment are frequently underrepresented, or explicitly excluded, in studies and trials targeting persons with hip fracture [18, 19]. With these exclusions, individuals may remain in acute settings with delays in discharge, where they are at risk of additional complications such as hospital-acquired infections [24], deconditioning [25], and mortality [16, 26], or are transitioned out of hospital without appropriate rehabilitation or support [27].

In this context, it is important to better understand what interventions currently exist and in what settings for people with hip fracture and co-occurring cognitive impairment in order to provide more evidence on rehabilitation and related outcomes for this population. To our knowledge, two similar systematic reviews have been conducted [28, 29]; however, there are limitations to these reviews that our scoping review aims to address. First, both reviews focused on specific healthcare sectors, such as community-based interventions [29], long-term care [28], and post-acute rehabilitation settings [28]. As current guidelines recommend mobilization within 24 to 48 hours post-surgery [20–22], it is important to examine interventions offered in acute care, as well as across the continuum of care (including post hospital care in the community). Secondly, the previous reviews limited their populations to older adults (≥65 years old). While older age is a risk factor for both hip fractures and cognitive impairment [30, 31], they can occur at earlier ages as well [32, 33].

The objective of this scoping review was to examine the extent, nature, and range of literature on rehabilitation interventions for adults (aged 18+) with hip fracture and cognitive impairment. A scoping review methodology was appropriate for addressing this goal as it allowed us to identify a broader range of literature available on this topic, examine characteristics pertaining to interventions for persons with hip fracture and cognitive impairment, address current gaps in the literature, and highlight areas that warrant future work.

## Methods

This scoping review was conducted based on the most recent methodology outlined by Peters and colleagues [34]. The Preferred Reporting Items for Systematic Reviews and Meta-Analyses extension for scoping review (PRISMA-ScR) was also followed (see S1 Table) [35].

### Protocol and registration

A protocol for this scoping review was registered on Open Science Framework (10.17605/OSF.IO/ZA92V).

### Eligibility criteria

The inclusion criteria for this scoping review were as follows: (1) included adults with hip fracture and cognitive impairment, (2) included a rehabilitation intervention that focused on at least physical functioning, and (3) published from January 1, 2000 to November 19, 2021. We only included articles published as of 2000 to ensure healthcare relevancy. Articles were excluded if they met any of the following criteria: (4) opinion pieces (e.g. editorial, commentary), (5) study protocols, (6) did not describe the rehabilitation intervention, or (7) conference abstracts.

### Information sources

Databases were selected based on their topic concentrations in order to ensure maximum recall of relevant studies [36]. The following electronic databases were searched on November 19, 2021: MEDLINE (Ovid Interface), EMBASE (Ovid Interface), CINAHL Plus (EBSCOhost Interface), APA PsycINFO (Ovid Interface), Cochrane Library, and Web of Science. The Physiotherapy Evidence Database (PEDro) was also searched for relevant randomized controlled trials, systematic reviews, and clinical practice guidelines. Using the final included articles, Web of Science was used to conduct forward and backward searching [37] on January 14, 2022.

### Search strategy

The original search strategy was developed by the research team. The search strategy underwent a peer review by a librarian using the Peer Review of Electronic Search Strategies (PRESS) checklist [38] and minor revisions were made (see S2 Table for the Medline search strategy). The search strategy used medical subject headings and keywords to combine three main concepts: hip fracture, cognitive impairment, and intervention. The search strategy was manually adapted for each database.

### Selection process

All articles from the database searches were imported into EndNote X8, and duplicates were removed following Bramer’s method for de-duplication [39]. Following de-duplication, articles were imported into Covidence for article screening. The titles and abstracts of 25 articles were screened by two reviewers (SJTG and LC) to test their agreement. The screeners had an agreement of 96%, so they proceeded with screening the remainder of the titles and abstracts. All articles were double screened and any disagreements were resolved through consensus. After the completion of the title and abstract screening, the same two reviewers (SJTG and LC) screened 15 full-texts to test their agreement. The screeners had an agreement of 93%, so they proceeded with screening the remainder of the full-text articles. All full-texts were double screened and disagreements were resolved through consensus.

### Data charting process

A study-specific, data extraction form was developed in Microsoft Excel to facilitate the consistent extraction of information. Two team members (SJTG and LC) tested the extraction form and completed a spot check of 10% of the articles to ensure the information extracted was complete, accurate, and consistent. Minor/no revisions were made to the data extraction form during this process.

### Data items

Data were extracted on general information (title, authors, journal, year of publication, funding), study characteristics (objective, research question, hypotheses, type of population, method of data collection, study design, theoretical orientation, eligibility criteria, primary and secondary outcomes, country, setting), rehabilitation intervention characteristics (description, content, frequency, duration, single or multi-component, format, modifications, tailoring, delivery, setting), population characteristics (sample size, age, sex, gender, ethnicity/race, income, education, marital status, household composition, employment status, reason for hospitalization, type/severity of cognitive impairment, comorbidities, residence pre-hospitalization), study outcomes and findings (results and key findings, conclusions), and qualitative findings, if applicable (themes, conceptualization of themes). The Template for Intervention Description and Replication (TIDieR) checklist was used to guide the data items extracted for rehabilitation intervention characteristics [40].

### Synthesis methods

Data were synthesized using descriptive approaches. We summarized the study types, years of publication, countries, populations, types of rehabilitation interventions, and outcomes of the interventions. We used content from the TIDieR checklist to guide the presentation of the results [40]. A critical appraisal of articles was not conducted, but is not a requirement for scoping reviews [35].

## Results

### Study selection

**Fig 1:**
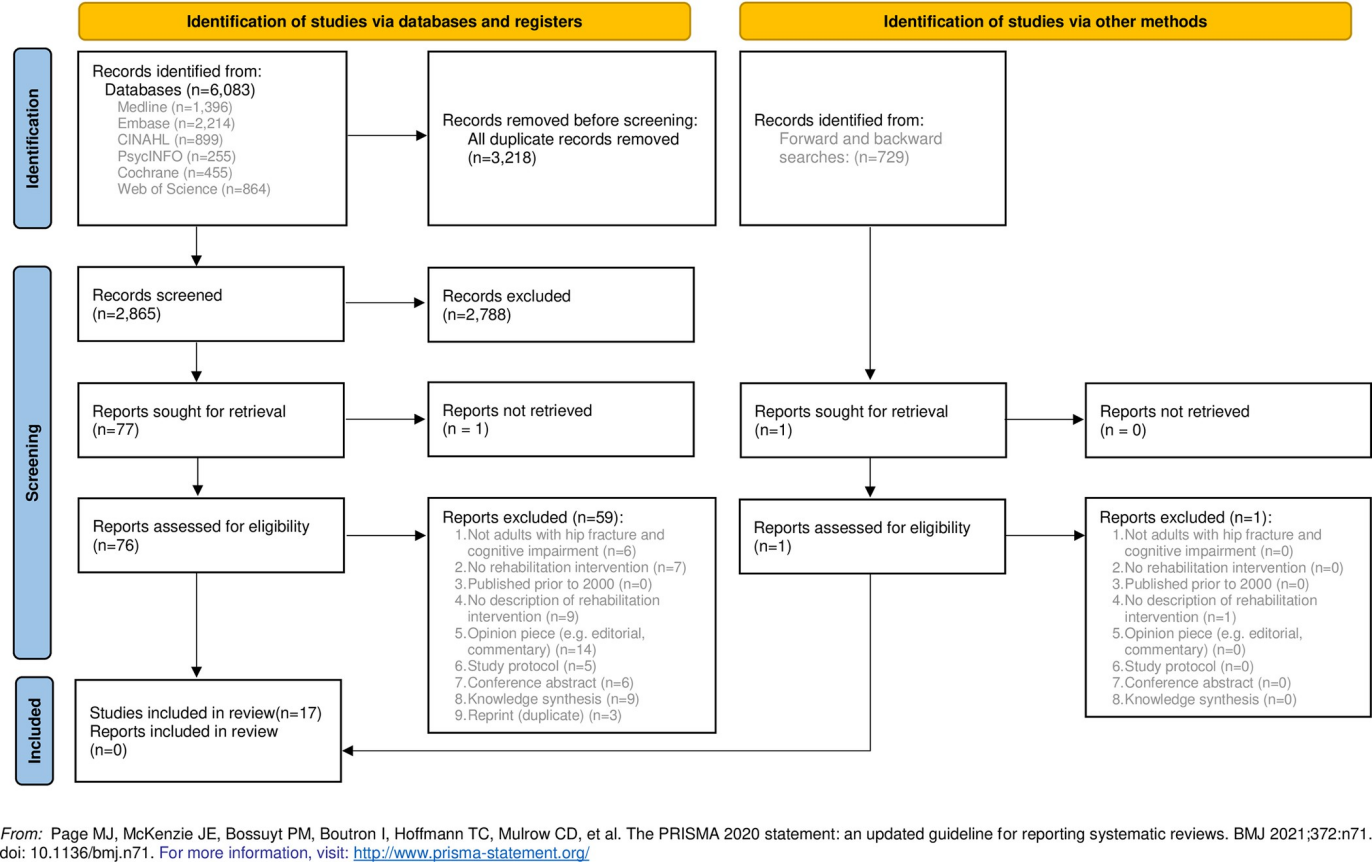
PRISMA 2020 flow diagram of included articles.

The database searches yielded 6,083 articles, which was reduced to 2,865 following deduplication (see Fig 1). During title and abstract screening, 2,788 articles were excluded. Of the 76 full-text articles screened, 26 met the eligibility criteria and were used to conduct the forward and backward searches. This resulted in an additional 729 titles and abstracts to screen; 728 were excluded, 1 was screened at the full-text level and excluded. We did not include the knowledge syntheses (systematic, scoping, and literature reviews; n = 9) in data extraction or synthesis, so 17 articles were included in this scoping review.

### Study characteristics

Table 1 displays the characteristics of the included articles. All of the studies but one (n = 16) used quantitative methods [41–48, 50–57]. Quantitative study designs were randomized controlled trials, including sub-analyses from the trials [43–46, 54–57], prospective and retrospective cohort studies [41, 42, 47, 50–53], and a quasi-experimental study [48]. Of these quantitative studies, three did not have a comparator. The one qualitative study used a case study design [49]. The majority of included articles were published from 2010 onwards (n = 14) [41, 42, 44–50, 53–57]. Studies were conducted in seven different countries: Sweden (n = 6) [41, 42, 44–46, 56], Taiwan (n = 3) [54, 55, 57], Canada (n = 3) [48, 49, 53], Finland (n = 2) [43, 51], Japan (n = 1) [47], Romania (n = 1) [50], and France (n = 1) [52].

**Table 1:** Characteristics of included articles (n = 17).

Author (year)	Country	Objective	Method Study Design	Participants	Sample Size	Key Conclusions
Al-Ani et al. (2010) [41]	Sweden	• To assess factors associated with activities of daily living and preserved walking ability at 4 and 12 months for persons with femoral neck fractures and cognitive impairment	Quantitative Observational Study	Older adults (65+) with femoral neck fracture and cognitive impairment	246 (19 patients died before discharge)	• Activities of daily living and preserved walking ability were associated with discharge to rehabilitation
Chammout et al. (2021) [42]	Sweden	• To identify the impact of postoperative geriatric rehabilitation on functional outcomes for elderly patients with femoral neck fracture and cognitive dysfunction	Quantitative Single centre prospective observational cohort	Patients (65+) with a displaced femoral neck fracture and cognitive dysfunction	98	• Significant decline in postoperative walking ability is associated with a lack of structured rehabilitation
Huusko et al. (2000) [43]	Finland	• To examine the effect of postoperative geriatric rehabilitation for elderly patients with hip fracture • To explore if patients with cognitive impairment benefit from geriatric assessments and rehabilitation	Quantitative Randomized, clinically controlled trial	Patients (65+) with hip fracture	243	• Active geriatric rehabilitation can facilitate the return to community for patients with hip fracture and mild to moderate dementia
Karlsson et al. (2016) [44]	Sweden	• To evaluate the effects of Geriatric Interdisciplinary Home Rehabilitation on older adults with hip fracture, including adults with cognitive impairment (walking ability, length of stay)	Quantitative Randomized controlled trial	Patients (70+) with cervical or trochanteric hip fracture	205	• Geriatric Interdisciplinary Home Rehabilitation can help improve short and long-term walking ability and reduce postoperative length of stay
Karlsson et al. (2020) [45]	Sweden	• To evaluate the effects of Geriatric Interdisciplinary Home Rehabilitation on older adults with hip fracture, including adults with cognitive impairment (independence in activities of daily living)	Quantitative Randomized controlled trial (subgroup analysis)	Patients (70+) with cervical or trochanteric hip fracture	205	• Geriatric Interdisciplinary Home Rehabilitation resulted in similar independence in activities of daily living when compared to in-hospital care and rehabilitation
Karlsson et al. (2020) [46]	Sweden	• To examine the effects of geriatric interdisciplinary home rehabilitation post hip fracture for adults with and without dementia • To describe the outcomes among adults with hip fracture and dementia	Quantitative Randomized controlled trial (subgroup analysis)	Patients (70+) with cervical or trochanteric hip fracture	205	• Geriatric Interdisciplinary Home Rehabilitation did not affect those with and without dementia differently • Dementia negatively impacted outcomes post hip fracture
Kazuaki et al. (2019) [47]	Japan	• To examine the effects of earlier, more frequent, and larger amounts of postoperative rehabilitation in hospital for patients with dementia and hip fracture (activities of daily living)	Quantitative Retrospective cohort study	Patients (65+) with dementia and hip fracture	43,206	• Improved recovery in activities of daily living was associated with more frequent, and larger daily amounts of postoperative rehabilitation
McGilton et al. (2013) [48]	Canada	• To examine the impact of a patient-centered rehabilitation model of care for older adults with cognitive impairment on mobility and probability of returning home	Quantitative Quasi-experimental design	Patients (65+) with hip fracture	149	• Older adults with cognitive impairment and hip fracture can be rehabilitated using an interdisciplinary, patient-centered rehabilitation model
McGilton et al. (2021) [49]	Canada	• To explore healthcare professionals perspectives on rehabilitation strategies for persons with cognitive impairment	Qualitative Case study	Healthcare professionals involved in rehabilitation of older adults with hip fracture and cognitive impairment	16	• Rehabilitation of persons with cognitive impairment is possible post hip fracture, but requires tailored approaches, learning, creativity, support, and ingenuity
Paul-Dan et al. (2019) [50]	Romania	• To examine postoperative weight-bearing following hip fracture for patients with dementia • To identify if weight-bearing is associated with future rehabilitation and 1-year mortality	Quantitative Retrospective Study	Patients (65+) with displaced femoral neck fracture and dementia	178	• Patients with total weight-bearing who were discharged to a rehabilitation facility had a better recovery (pre-fracture level) and one-year survival rate
Raivio et al. (2004) [51]	Finland	• To examine to impact of weight-bearing restrictions on length of rehabilitation time • To explore if patients with dementia had difficulty following weight-bearing restrictions	Quantitative Retrospective Study	Patients with hip fracture	98	• Strict weight-bearing restrictions may impair rehabilitation outcomes and may be more severe for patients with dementia
Rolland et al. (2004) [52]	France	• To examine the effect of cognitive status on functional gain for patients in a geriatric rehabilitation unit	Quantitative Prospective study	Patients (70+) hospitalized for rehabilitation after hip fracture surgery	61	• Patients with hip fracture (with and without cognitive impairment) can benefit from rehabilitation programs
Seitz et al. (2016) [53]	Canada	• To examine associations between access to postoperative rehabilitation and long-term care admission, mortality, and risk of repeat falls and fractures	Quantitative Retrospective cohort study	Individuals with dementia and hip fracture	11,200	• Postoperative rehabilitation was associated with decreased risks of long-term care placement and mortality
Shyu et al. (2012) [54]	Taiwan	• To evaluate the effects of an interdisciplinary program post hip fracture	Quantitative Randomized controlled trial (post-hoc analysis)	Patients (60+) with accidental single‐side hip fracture	160	• Walking ability and physical function improved for patients with cognitive impairment
Shyu et al. (2013) [55]	Taiwan	• To investigate the 2-year trajectory of patients with hip fracture and cognitive impairment • To assess the effects of an interdisciplinary program on level and speed of change of cognitive function, as well as the impact of the cognitive function on trajectory	Quantitative Randomized controlled trial	Patients (60+) with accidental single‐side hip fracture	160	• Long-term postoperative cognitive functioning improved
Stenvall et al. (2012) [56]	Sweden	• To investigate the effects of a multidisciplinary postoperative program on complications and functional recovery	Quantitative Randomized controlled trial (subgroup analysis)	Patients (70+) with femoral neck fracture	64	• Patients with femoral neck fracture and dementia can benefit from multidisciplinary postoperative programs
Tseng et al. (2021) [57]	Taiwan	• To develop and test a family-centered model of care (self-care ability, nutritional status, health related quality of life, and self-rated health)	Quantitative Randomized controlled trial	Older persons (60+) with hip fracture and cognitive impairment	152	• Physical recovery of patients with hip fracture and dementia did not improve, but caregivers’ self-efficacy and competence was improved

### Population characteristics

The majority of studies focused on and included older adults (defined as 60, 65, or 70 years and older) [41–48, 50, 52, 54–57] and all but one study [49] had participants with a mean age of 75 years or older. Six articles included only participants with cognitive impairment and hip fracture [41, 42, 47, 50, 53, 57], while the remaining 11 articles included both those with and without cognitive impairment [43–46, 48, 49, 51, 52, 54–56]. The Mini Mental State Examination (MMSE) was most commonly used to assess cognitive status followed by the Short Portable Mental Status Questionnaire (SPMSQ). Functional status was assessed using a number of measures including: ASA Physical Status Classification System, Charnley classification, independent walking ability, Barthel Activities of Daily Living (ADL) Index, Katz ADL Index, and Functional Independence Measure. All articles reported the sex or gender of the participants, but none reported both. Participant characteristics that were not consistently collected across the included articles were education level, marital status, household composition, and employment status. The race, ethnicity, and income level of participants were not reported in any article.

### Intervention characteristics

Table 2 displays the intervention characteristics of the included articles. About half of the interventions (n = 8) were specific to physical rehabilitation [41–43, 47, 50–53], while the other half (n = 9) incorporated content into the intervention that addressed additional aspects of the patients’ care journey or support (e.g. discharge planning, patient and family education, nutrition) [44–46, 48, 49, 54–57]. The physical rehabilitation component of the interventions most commonly incorporated standing, walking with or without support, range of motion, balance exercises, and functional strength. As measured by the primary or secondary outcomes, the focus of the majority of interventions was to improve participants’ physical functioning (walking ability) and ability to perform activities of daily living [41, 42, 44–48, 50–52, 54, 56, 57]. Other outcomes assessed less frequently were mortality [42, 43, 46, 50, 53, 54, 56], length of stay [43–46, 56], readmissions [45, 46, 54, 56], and quality of life [42, 57]. The experiences of patients and families were not qualitatively explored in any of the included articles. The interventions were most commonly delivered by an interdisciplinary team consisting of a combination of the following: physiotherapists, occupational therapists, nurses, social workers, physicians and/or geriatricians. Of the two interventions that were delivered by a single profession, physiotherapists were responsible [42, 50]. About half of the articles (n = 9) described modifying or tailoring the intervention to the individuals’ needs [42–46, 49, 52, 54, 55]. This was usually dependent on how the patient was progressing with their physical rehabilitation [43–46, 52, 54]; however, one article described modifications or adaptations that were made for individuals depending on their level of cognitive impairment [42]. All interventions were initiated face-to-face in hospital (in acute care or inpatient rehabilitation) and six included cross-sectoral components [44–46, 54, 55, 57] in the form of in-home rehabilitation [44–46, 54, 55, 57], in-home training for families [57], community and long-term care assessments, referrals, or initiation of services [54, 55, 57], and telephone follow-ups [54, 55]. There was wide variation in the length, frequency, and duration of the interventions. The length of physical rehabilitation sessions varied from 20 minutes to 60 minutes, the frequency varied from multiple sessions daily to a few sessions per week, and the duration was not consistently reported (some were only delivered until discharge from hospital, while others included follow-up in the community). Most articles described starting physical rehabilitation one day post-surgery.

**Table 2:** Intervention characteristics (n = 17).

Author	Intervention Description	Target Population	Setting	Delivery	Frequency and Duration	Tailoring/ Modification	Results
Al-Ani et al. (2010) [41]	***Rehabilitation*** To restore patients’ walking ability and allow them to return to their pre-injury living arrangements	Older adults (65+) with femoral neck fracture and cognitive impairment	In hospital	Physio and occupational therapists	Several days–specific frequency not reported From surgery to discharge, with follow-up at 4 and 12 months	Not reported	• Walking ability at 12-months was significantly associated with discharge to a rehabilitation unit and pre-fracture walking ability • Preserved activities of daily living index at 12-months was significantly associated with discharge to a rehabilitation unit and pre-fracture index level
Chammout et al. (2021) [42]	***Geriatric Rehabilitation*** To restore walking ability to the patients’ pre-fracture level prior to discharge	Patients (65+) with a displaced femoral neck fracture and cognitive dysfunction	In hospital (geriatric ward)	Physiotherapists	Not reported Usual stay was 10 days	Rehabilitation on the geriatric ward was individually adapted based on cognitive dysfunction	• Geriatric rehabilitation was correlated with improved outcomes and decreased likelihood of being confined to a wheelchair or bedridden at one-year • One year mortality rate was 31%
Huusko et al. (2000) [43]	***Geriatric Ward*** To deliver physio and occupational therapy, conduct joint meetings (with discharge planning), promote early ambulation, self-motivation, and functional ability, and conduct patient/family counselling	Patients (65+) with hip fracture	In hospital (geriatric ward)	Geriatric team (geriatrician internist, general practitioner, nurses with geriatric training, social worker, neuro-psychologist, physio and occupational therapists, consultant, neurologist, psychiatrist)^1^	Weekly team meetings and physio 2x/day Median length of stay was 18 days	Methods for improving rehabilitation were discussed in weekly meetings (nurses and physiotherapists)	• Median length of stay was shorter for patients with hip fracture and moderate dementia in the intervention group compared to the control (47 vs 147 days) • 3 months post-surgery, more patients with mild and moderate dementia (91% and 63%) were living independently compared to the control group (67% and 17%) • No significant differences in mortality or length of stay for patients with severe dementia
Karlsson et al. (2016) [44]	***Geriatric Interdisciplinary Home Rehabilitation*** To promote early discharge from hospital and continue rehabilitation in participants’ homes, with a focus on detection, prevention and treatment of postoperative complications	Patients (70+) with cervical or trochanteric hip fracture	In hospital and community (geriatric ward, ordinary housing, and residential care facilities)	Nurse, physio and occupational therapists, geriatrician, social worker, dietician	Post discharge: ~1x/day home visit and then tailored based on needs Maximum of 10 weeks	Rehabilitation was individually designed for patients’ goals	• No significant differences between the intervention and control groups at 3 and 12 months for walking ability, use of walking device, and gait speed • Median postoperative length of stay in the geriatric ward was significantly shorter (6 days) for the intervention group
Karlsson et al. (2020) [45]	***Geriatric Interdisciplinary Home Rehabilitation*** To promote improved physical function, prevent falls, modify the home environment, and provide training in physical and instrumental activities of daily living and use of assistive devices	Patients (70+) with cervical or trochanteric hip fracture	In hospital and community (geriatric ward, ordinary housing, and residential care facilities)	Nurse, physio and occupational therapists, geriatrician, social worker, dietician	Median 21 days of intervention and 14 home visits Maximum of 10 weeks	Rehabilitation and the number of home visits were individually tailored	• No significant differences in performance of activities of daily living between the intervention and control groups • Both groups recovered comparably to their pre-fracture level of independence
Karlsson et al. (2020) [46]	***Geriatric Interdisciplinary Home Rehabilitation*** To promote improved physical function, prevent falls, modify the home environment, and provide training in physical and instrumental activities of daily living and use of assistive devices	Patients (70+) with cervical or trochanteric hip fracture	In hospital and community (geriatric ward, ordinary housing, and residential care facilities)	Nurse, physio and occupational therapists, geriatrician, social worker, dietician	Median 17 days of intervention Maximum of 10 weeks	Rehabilitation and the number of home visits were individually tailored	• Falls and mortality were comparable in both groups (intervention vs. usual care) • Activities of daily living and walking ability were comparable regardless of cognitive status (dementia vs. not) • Median postoperative length of stay was shorter for patients in the intervention group (18 days vs 23 days)
Kazuaki et al. (2019) [47]	***Intensive In-Hospital Rehabilitation*** No description	Patients (65+) with dementia and hip fracture	In hospital	Not reported	20 minutes of rehabilitation, maximum of 9x/day Median length of stay was 21 days	Not reported	• Delayed rehabilitation was significantly associated with lower activities of daily living at discharge • More frequent, higher frequencies, and larger amounts of rehabilitation were significantly associated with increased activities of daily living at discharge
McGilton et al. (2013) [48]	***Patient-Centered Rehabilitation Model Of Care For Older Adults With Cognitive Impairment (PCRM-CI)*** To provide interdisciplinary rehabilitation to patients through rehabilitation management, dementia management, delirium management, staff education and support, and family education and support	Patients (65+) with hip fracture	In hospital (inpatient musculoskeletal unit)	Advanced practice nurse with gerontological expertise, unit staff	Not reported Not reported	Not reported	• No differences in mobility gain were identified between the groups • Patients in the intervention group were significantly more likely to return home post-discharge than usual care
McGilton et al. (2021) [49]	***Patient-Centered Rehabilitation Model Of Care For Older Adults With Cognitive Impairment (PCRM-CI)*** To provide interdisciplinary rehabilitation to patients through rehabilitation management, dementia management, delirium management, staff education and support, and family education and support	Healthcare professionals involved in rehabilitation of older adults with hip fracture and cognitive impairment	In hospital (inpatient musculoskeletal unit)	Physio and occupational therapists, nurse, dietician, social worker, geriatrician, physician, advanced practice nurse	PT/OT 1x/day for 1 hour, 5 days/week Not reported	A tailored approach was noted as an essential component of rehabilitation	• Essential components of rehabilitation for adults with cognitive impairment include staff education and support, tailored approaches and partner involvement
Paul-Dan et al. (2019) [50]	***Physical Therapy—Weight Bearing*** To provide intensive physical therapy through passive, active-assisted, and active sessions (gait training, walking, stair climbing)	Patients (65+) with displaced femoral neck fracture and dementia	NR	Physical therapist	PT 30–40 minutes 1-2x/day during the week and 1x/day on weekends and holidays Not reported	Not reported	• Patients with immediate total weight bearing and those discharged to rehabilitation had an enhanced return to pre-fracture level of independence and lower rates of one-year mortality
Raivio et al. (2004) [51]	***Physical Therapy—No Weight Bearing Restriction*** To provide physical therapy and guided exercises focused on strengthening, walking, and balance training	Patients with hip fracture	In hospital	Nurse, physical therapist	30 minutes 1x/day, 5x/week Average 38.4 days	Not reported	• Rehabilitation time was longer for patients with weight-bearing restrictions than those without
Rolland et al. (2004) [52]	***Geriatric Rehabilitation Unit*** To establish goals for the patient, organize the rehabilitation program, and assess the results	Patients (70+) hospitalized for rehabilitation after hip fracture surgery	In hospital (geriatric rehabilitation unit)	Physiotherapist, geriatrician, physiotherapist, psychologist, geriatric nurse	1 hour, 2x/day, 5x/week Not reported	Patient goals, the rehabilitation program, and results were discussed in weekly meetings	• Patients with cognitive impairment had lower functional independence measures at admission and discharge • Cognitive status was not significantly associated with functional gain • Functional gain was insignificant between the groups and was related to baseline functional status
Seitz et al. (2016) [53]	***Rehabilitation*** Three types of rehabilitation settings: complex continuing care (low-intensity, long duration), home care rehabilitation (in-home physio and occupational therapy), inpatient rehabilitation (highest intensity)	Individuals with dementia and hip fracture	In hospital and community (complex continuing care–hospital; inpatient rehabilitation–hospital; home care rehabilitation–home	General medical care, physio and occupational therapists, nurses	Complex continuing care: PT/OT 2-3x/week 8–12 weeks Inpatient rehabilitation: PT/OT up to 5x/week 4–6 weeks Home care rehabilitation: 1-2x/week 6–8 weeks	Not reported	• Of those with dementia and hip fracture, 40% did not receive rehabilitation, 22% were admitted to complex continuing care, 10% received home care rehabilitation and 27% inpatient rehabilitation • All types of rehabilitation were associated with a lower risk of mortality than no rehabilitation • Inpatient and home care rehabilitation were associated with a lower risk of long-term care admission post-discharge compared to no rehabilitation
Shyu et al. (2012) [54]	***Interdisciplinary Intervention*** To provide a geriatric consultation service, a rehabilitation program, and a discharge‐planning service	Patients (60+) with accidental single‐side hip fracture	In hospital and community	Geriatrician, geriatric nurses, rehabilitation physician, physical therapist	Inpatient: geriatric nurse 4 visits, PT 2 visits, physician 1 visit In-home: 4x/months for 3 months, PT 3 visits Average stay was 10.1 days	Modified care plans were developed based on pre and post-surgical team assessments	• Among patients with cognitive impairment, more in the intervention groups regained their pre-fracture walking ability, performance in activities of daily living, and were readmitted to hospital than in the control group • Among patients without cognitive impairment, more in the intervention group regained their pre-fracture walking ability, and had fewer falls and emergency room visits than in the control group
Shyu et al. (2013) [55]	***Interdisciplinary Intervention*** To provide a geriatric consultation service, a rehabilitation program, and a discharge‐planning service	Patients (60+) with accidental single‐side hip fracture	In hospital and community	Geriatrician, geriatric nurses, rehabilitation physician, physical therapist	Not reported 3 months	Exercise protocol was individualized for each patient	• Patients in the intervention groups were 75% less likely to be cognitively impaired at 6 months post-discharge (than usual care)
Stenvall et al. (2012) [56]	***Multidisciplinary Intervention Program*** To provide comprehensive geriatric assessments and rehabilitation through detection, prevention, and treatment of delirium, falls, pain, pressure ulcers, and malnutrition	Patients (70+) with femoral neck fracture	In hospital (orthopedic department)	Geriatric team (physician, nurse, physio and occupational therapists, care staff)	Not reported Not reported	Not reported	• Significantly fewer postoperative complications (urinary tract infections, nutritional problems, delirium, falls) in the intervention group • A greater proportion of patients in the intervention group regained their pre-fracture walking ability • A greater proportion of patients in the intervention group regained their pre-fracture level of activities of daily living
Tseng et al. (2021) [57]	***Family Centered Care Model*** To provide family-centered care through geriatric assessments, discharge planning, in-home rehabilitation, and family caregiver-training for dementia care	Older persons (60+) with hip fracture and cognitive impairment	In hospital and community	Geriatrician, geriatric nurses, rehabilitation physician, physical therapist	In hospital: geriatric nurse visit 1x/day In-home rehab: 1x/week, then tapered 12 months	Not reported	• Patients in the intervention group had a greater rate of improved self-rated health and nutritional status • Caregivers in the intervention group had a higher level of competence and greater rates of improved competence and self-efficacy

^1^ The geriatric team collaborates with patients, families, local health centres, nursing homes, home help, and home care

### Intervention outcomes

The outcomes of the interventions varied across the included articles. Improved walking ability was identified in four articles [41, 42, 44, 56]; however no differences were noted in three articles [44, 46, 48]. Preserved or improved performance of activities of daily living (e.g. mobility, bathing, dressing, toileting and continence, transferring, feeding) was identified in four articles [41, 47, 54, 56], with no differences found in two [45, 46]. Length of hospital stay or rehabilitation time was shorter for the intervention group, compared to the control group in five articles [43, 44, 46, 51, 56]. Lower rates of mortality in intervention groups compared to the control were found in two articles [50, 53], with no differences identified in three articles [43, 46, 56]. Four of the included articles compared outcomes between those with hip fracture and cognitive impairment and those without cognitive impairment [43, 46, 52, 54]. Three of the four studies found comparable outcomes between the groups [43, 46, 52]; activities of daily living and walking ability were comparable regardless of cognitive status [46], functional gain was not associated with cognitive status [52], and the ability to return to independent living was comparable between patients with mild cognitive impairment and those with normal cognitive function [43]. The article that found differences noted that patients with cognitive impairment in the rehabilitation group did not experience improvements in subsequent falls (fewer falls) or emergency room visits, as those without cognitive impairment experienced [54]. Patients with cognitive impairment (in the control group) also had poorer outcomes with walking ability and activities of daily living performance when compared to those without cognitive impairment (in the control group) [54].

## Discussion

The purpose of this scoping review was to identify rehabilitation interventions for adults with hip fracture and cognitive impairment, while not limiting by sector of implementation or age of the population. Based on the 17 included articles, we found that (1) several forms of rehabilitation interventions were available, with varying outcome measures and success; however, none of the included studies explored patient and family experiences; (2) information on how to adapt rehabilitation interventions for individuals with cognitive impairment was lacking; and (3) few interventions were implemented across sectors.

Sixteen of the 17 included articles were quantitative and most commonly assessed participants’ physical functioning (walking ability), ability to perform activities of daily living, mortality, length of stay, readmission rates, and quality of life. Despite varying results across these outcomes, we identified some evidence to suggest that patients with cognitive impairment should not be excluded from rehabilitation. For example, improvements were identified in walking ability, activities of daily living, length of stay, and physical functioning, and in some cases, the improvements were comparable to those seen in individuals without cognitive impairment. This echoes findings from previous research [28, 29, 58], including two systematic reviews in which benefits of rehabilitation interventions for older adults with hip fracture and cognitive impairment were reported [28, 29], as well as noting that participants did not experience harm (e.g., falls, exacerbation of previous medical issues) when taking part in rehabilitation activities [28]. Further to these reviews, a qualitative study conducted by Sondell and colleagues described the benefits of a multidimensional and interdisciplinary rehabilitation program for older adults with dementia, which included: improved physical abilities, motivation and self-efficacy, feelings of empowerment, the ability to participate in everyday activities, an increased sense of responsibility to continue exercise post-rehabilitation, and the creation of friendships [58]. The study by Sondell et al. provided important contextual information on how the participants experienced the multidimensional interdisciplinary rehabilitation in dementia program, which is currently missing for adults with hip fracture and cognitive impairment. This presents a critical area for future research to explore, in order to better understand the experiences, perceptions, and reflections of those with lived experience pertaining to current rehabilitation interventions.

This scoping review also identified the need to better understand how to modify and tailor interventions for individuals’ needs, especially how to adapt interventions for those with differing levels of cognitive impairment. Only one of the included articles explicitly reported adapting the intervention based on individuals’ cognitive impairment; however, the process for doing so was not described. This finding is similar to that of Chu and colleagues, who described the need for an increased focus on rehabilitation interventions that are tailored, or potentially newly developed, for patients with cognitive impairment [29].

Opportunities to better understand how to tailor rehabilitation interventions to those with hip fracture and cognitive impairment can be explored through qualitative research and co-design. Qualitatively exploring the perspectives and experiences of patients, caregivers, providers, and organizational leaders can serve as a foundational starting point for better understanding patient and family needs during rehabilitation post-hip fracture. Based on their experiences, individuals can provide valuable insights into what is working well, what can be improved, as well as new ideas for tailoring programs for those with cognitive impairment. Additionally, rehabilitation interventions would benefit from being co-designed in collaboration between patients, families, and providers. Since none of the included articles explored patient and caregiver experiences with the interventions, co-design allows for the integration of these perspectives. Co-design includes the meaningful involvement of stakeholders during the planning, design, implementation, and adaptation of the intervention in order to meet the needs and preferences of its users [59]. Despite not having a standardized process, core principles of co-design include: equality, openness, respect, empathy, understanding, and improvement [60, 61]. Importantly, co-design offers a number of benefits for all stakeholders such as increased buy-in, enhanced understanding of goals and objectives, and improved experiences [59–61].

All of the interventions were initiated in-hospital, in acute care or inpatient rehabilitation, with only six including cross-sectoral components. Following a hip fracture, patients frequently experience numerous transitions across different settings and healthcare providers [62, 63]. Transitions in care have been identified as a vulnerable time for patients and families, often characterized as fragmented and resulting in poor health outcomes, including deconditioning, decreased satisfaction, high readmission rates, increased adverse events, and unmet needs [64, 65]. Despite the potential for poor health and social outcomes during care transitions, we found that only half of the articles included in this review incorporated components into the intervention that extended beyond physical rehabilitation to address rehabilitation more holistically (discharge planning, nutrition, and patient and family education). The connection between physical health, mental health, and social health has been well-documented [66, 67], but the integration of the three into rehabilitation interventions for adults with hip fracture and cognitive impairment is lacking. Based on the potential negative consequences that can occur during care transitions, rehabilitation interventions should be multidimensional, addressing physical, mental, and social health, and include cross-sectoral components to ensure continuity along the continuum of care (hospital, primary care, rehabilitation, home and community care) for adults with cognitive impairment following a hip fracture.

### Gaps and opportunities for future research

This scoping review identified several gaps in the literature that warrant additional research. First, patient, family, and provider experiences and perspectives should be explored during the development, implementation, and evaluation of interventions for persons with cognitive impairment and hip fracture. Second, rehabilitation interventions should be co-designed with patients and families to ensure their insights and experiences can be used to inform programs and practiced-based decisions. Lastly, based on the interconnectedness of physical, mental, and social well-being [66, 67], there is a need to incorporate components into rehabilitation interventions that extend beyond improving physical functioning (i.e., social aspects, mental health, education for patients and families) and are implemented across sectors.

### Strengths and limitations

A few notable strengths of this scoping review are working in collaboration with a librarian to develop a comprehensive search strategy, undergoing a peer review of the search strategy, and supplementing the search with forward and backward searching. Additionally, we used a rigorous double-screening process to ensure two individuals independently screened all potential articles. Despite these strengths, it is possible that some relevant articles were missed due to only searching literature published from 2000 onwards and our search being in English.

## Conclusions

This scoping review identified a number of rehabilitation interventions for adults with hip fracture and cognitive impairment. The majority of included studies were quantitative, with a lack of exploration of patient and family experiences. Interventions had varying outcomes, but there were some positive results, highlights the need for providing post-hip fracture rehabilitation to adults with cognitive impairment. All interventions were initiated in hospital, with few including cross-sectoral components. Future work should focus on exploring patient, family, and provider experiences with rehabilitation interventions, tailoring interventions for those with cognitive impairment, and implementing interventions across sectors.

## Supporting information

S1 Table Preferred Reporting Items for Systematic reviews and Meta-Analyses extension for Scoping Reviews (PRISMA-ScR) checklist. (DOCX)

S2 Table Medline search strategy. (DOCX)
