# Supplementary material for: Rehabilitation interventions for persons with hip fracture and cognitive impairment: A scoping review
Source: PLoS One. 2022 Aug 15;17(8):e0273038. doi: 10.1371/journal.pone.0273038 (PMC9377630; doi:10.1371/journal.pone.0273038)
Supplement: S2 Table — (DOCX) [file pone.0273038.s002.docx]

# **Supporting information**

S2 Table. Medline Search Strategy

| **#** | **Search Term** |
| --- | --- |
| 1 | exp Hip Fractures/ |
| 2 | ((Hip* or femur* or femoral* or pertrochant* or intertrochant* or subtrochant* or trochant* or extracapsular* or orthop*) adj3 (fracture* or crack* or broke* or break*)).tw,kf. |
| 3 | 1 or 2 |
| 4 | Hip/ |
| 5 | Fractures, Bone/ |
| 6 | 4 and 5 |
| 7 | 3 or 6 |
| 8 | exp Dementia/ or exp Delirium/ or Neurocognitive Disorders/ or exp Cognition Disorders/ or Intellectual Disabilities/ or Auditory Perceptual Disorders/ |
| 9 | ((Cogniti* or Neurocognitive) adj2 (impair* or declin* or loss* or disorder* or dysfunction*)).tw,kf. |
| 10 | (Dement* or Alzheimer* or deliri* or amnesia* or Huntington* or Creutzfeldt* or Lewy bod* or Kluver-Bucy* or aphasia* or palsy* or (auditory adj3 disorder*)).tw,kf. |
| 11 | ((mental adj2 deficienc*) or (intellectual adj3 (disorder* or disabilit))).tw,kf. |
| 12 | 8 OR 9 or 10 or 11 |
| 13 | Health Plan Implementation/ or Delivery of Health Care/ or health care reform/ or patient care management/ or critical pathways/ or practice guideline as topic/ or health policy/ or Delivery of Health Care, Integrated/ or evidence-based practice/ or psychosocial intervention/ or exp Psychotherapy/ or Internet/ or Internet-based intervention/ or Telemedicine/ or early medical intervention/ or exp rehabilitation/ or exp physical therapy modalities/ or exp Musculoskeletal manipulations/ |
| 14 | ((internet or online or web?based or virtual) adj3 intervention*).tw,kf. |
| 15 | ((telemedicine or mhealth or ehealth or mobile) adj2 health).tw,kf. |
| 16 | (strateg* or intervention* or program* or service* or model* or initiative* or polic* or plan* or re?design* or design* or tool* or system* or guideline* or rehabilitat* or therap* or (best adj2 practice*)).tw,kf. |
| 17 | ((health adj2 implementation) or (health?care adj3 deliver*) or (patient adj2 management) or (health adj2 implementation) or (health?care adj2 reform*) or ((care or patient or health?care) adj3 transition*) or ((clinical or critical) adj2 path*)).tw,kf. |
| 18 | 13 or 14 or 15 or 16 |
| 19 | 7 and 12 and 18 |
| 20 | limit 19 to yr="2000 -Current" |
